# Towards a Sustainable Healthcare System: A Concept Paper for a National Hybrid Financing Model for Malaysia

**DOI:** 10.34172/ijhpm.9223

**Published:** 2025-09-14

**Authors:** Mohd Arshil Moideen, Wan Nadiah Wan Mohd Abdullah Yaakob, Khasnur Abd Malek

**Affiliations:** ^1^Jeffrey Cheah School of Medicine and Health Sciences, Monash University Malaysia, Subang Jaya, Malaysia.; ^2^Independent Researcher, Kuala Lumpur, Malaysia.; ^3^Primary Care Medicine Department, Faculty of Medicine, Universiti Teknologi MARA, Selangor, Malaysia.

## Introduction

 This concept paper for a hybrid healthcare financing system directly responds to the Malaysia Madani Government’s call for comprehensive, conceptual frameworks to diversify funding for public healthcare services.^1^ While the government has made serious attempts at reform, including the proposed National Health Insurance under the broader National Health Financing Scheme,^2^ no concrete steps have followed. This concept paper attempts to deliver an implementable framework for actions to bridge the gap.

 Malaysia operates a dual healthcare system, a government-funded public sector and a fee-for-service private sector.^3^ The public system is largely subsidized through government revenue, without mandatory national health insurance contributions. As a result, residents pay only minimal fees for services at public clinics and hospitals. However, persistent budget deficits have gradually shifted more of the financial burden onto the public, driving up out-of-pocket (OOP) healthcare expenditure.^4^ During economic downturns, this trend risks further cuts to essential health services.

 This concept paper is not intended to replace the current healthcare financing system. Instead, it aims to introduce a complementary National Social Health Scheme (NSHS), a hybrid financing model designed to generate a stable and sustainable revenue stream for services delivered not only in public healthcare settings but also through public-private partnerships (PPPs). This involves strategic engagement private general practitioners (GPs) for screening and preventive care, and private hospitals to absorb overflow cases arising from long wait times, limited capacity, or service quality gaps at public hospitals. This hybrid model eases fiscal pressure and improves both access and quality of care through shared responsibility.

## Urgent Need to Reinvent Current Healthcare Financing System

 Even with the Malaysian Madani government’s 11.5% increase in healthcare spending, from RM32.41 billion in 2022 to RM36.1 billion in 2023,^5^ the system continues to struggle against rising medical inflation, which has consistently outpaced general inflation over the past decade. In 2024, Malaysia’s medical inflation rate stood at 11.9%, one of the highest in the region compared to Singapore (9.5%), Indonesia (10.1%), and Thailand (7.1%).^6^ This surge is driven by escalating costs of pharmaceuticals, medical equipment, and operational expenses; an ageing population; the introduction of new treatment modalities^6^; and insurers’ pursuit of profit margins.^7^ While most private hospitals operate on profit margins below 10%,^8^ their charges still contribute significantly to overall inflation.

## Malaysia’s High Household Out-of-Pocket Healthcare Expenditure Burden

 Rising healthcare costs, with underfunded public provision and coverage gaps in private insurance, have forced the public healthcare system to rely on lower-cost drugs and shift the cost of implants, advanced medications, and medical devices onto patients.^9^ Consequently, OOP reached RM22.38 billion in 2022, an equivalent to 1.64% of Malaysia’s gross domestic product (GDP),^9^ and is projected to grow by 0.39% annually.^10^ This trend indicates that Malaysians are either choosing, or being compelled, to pay directly for healthcare, placing a significant financial strain on families.

## Healthcare Coverage Fall Short for the Vulnerable Group

 The bottom 40% of earners (B40) qualify for the fully subsidised PeKa B40 scheme,^11^ but its basic screenings and limited follow-up care fail to meet real healthcare needs.^12^ This is further limited with only 10% uptake and few GPs involvement. PeKa B40 must be replaced with comprehensive coverage, to ensure the B40 are not left behind.

## Crisis in Care: Malaysia’s Medical Shortage

 Malaysia’s public healthcare system serves most of the population,^3^ is critically understaffed, especially in specialist care, leading to long waits, overcrowding, and declining quality.^13^ The national doctor-to-patient ratio of 1:417 masks the true shortage in the public sector, as it counts doctors from private, university, and defence institutions as well.^14^ At the core of the crisis is a decline in new medical officers, driven by low pay, job insecurity, limited career prospects, and tough working conditions.^5^ If unresolved, this talent drain will accelerate, further weakening the public healthcare system and pushing more citizens toward costly private care.

## Simply Increasing Government-Funded Healthcare Fund Is Not the Solution for Now

 Malaysia’s total healthcare expenditure is low at 4.4% of GDP, well below the 5.2% to 9.3% seen in Organisation for Economic Co-operation and Development countries such as Thailand, Singapore, and Australia.^10^ While there is scope to expand the tax base, for example by reintroducing the Goods and Services Tax and raising sin taxes on tobacco and alcohol, a significant GDP allocation increase is unrealistic for now. Poorly integrated electronic medical record systems hamper seamless care and accurate data tracking and without reliable data, more spending will not improve outcomes,^15^ as we cannot fix what we cannot measure. As data and efficiency improve, the complementary NSHS can provide vital financial support to strengthen healthcare delivery.

## Why Malaysia Must Shift to a Hybrid Healthcare Financing Model

 Malaysia can no longer rely solely on a fully government-funded system. Tax-funded models offer equity but are vulnerable to downturns, risking rationing and delays.^16^ Hybrid models, as in Australia and Singapore,^17^ balance public funding with individual contributions, enabling faster access, broader care, and financial resilience. With rising non-communicable diseases, an aging population, and growing healthcare demand, Malaysia must urgently adopt a hybrid system to share responsibility, ensure sustainability, and expand the continuum of care.

## Description of a Hybrid Healthcare System

###  Funding Resources 

 This hybrid healthcare financing system (Figure) rests on two primary resources, the NSHS and Government fund. The NSHS is intended to be voluntary, not mandatory, will consolidate contributions into a single national pool to fund a healthcare package provided at the public and private primary care centre for screening and preventive care and at public hospitals for secondary care. When public hospital capacity is exceeded, especially for urgent care or time sensitive care, pre-qualified private hospital providers will be engaged. These services will be funded by NSHS allocations, ensuring uninterrupted care at no extra cost to patients. Table illustrates the proposed model for effective care delivery.

**Figure:**
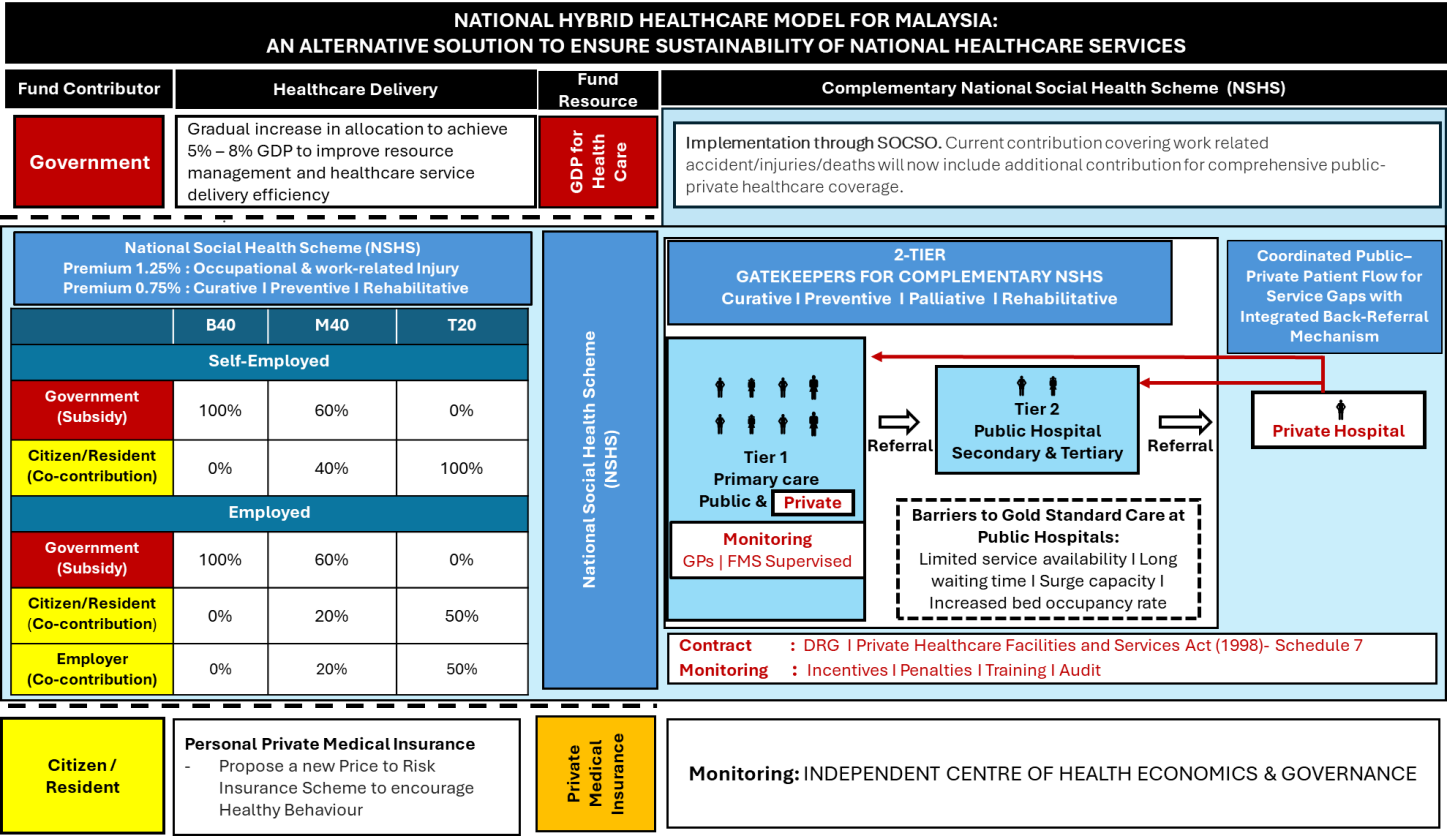
The Framework for the Proposed National Hybrid Healthcare Financing System. Copyright ID: CRDV2025W06807 (2025). Abbreviations: GDP, gross domestic product; B40, the bottom 40% of income earners; M40, the middle 40% of income earners; T20, the top 20% of income earners; DRG, diagnostic-related groups; SOCSO, Social Security Organization; GPs, general practitioners.

**Table:** Case Simulations Illustrating the Impact of National Social Health Scheme and Public-Private Partnership Synergy in Healthcare Delivery

**Case Simulation**	**Profile**	**NSHS Contributions**	**Incident**	**NSHS Implementation for Improved Healthcare Delivery**	**Key Synergy**
Case 1	Mr. A is a B40 Malaysian citizen.	Mr. A’s contributions to the NSHS are fully subsidized by the government.	After sustaining injuries in an accident, Mr. A required surgery involving an implantation device costing RM8000.	The NSHS fully covered the cost, enabling the procedure to be performed at a public hospital without delay. Post-surgical rehabilitation is critical to restore function and prevent long-term disability. However, due to extended wait times in the public rehabilitation system, Mr. A faced delays in accessing therapy. To ensure timely intervention, the government utilized NSHS funds to contract the rehabilitative services to an accredited private provider.	Mr. A’s case illustrates the power of NSHS to close critical care gaps, by delivering timely, outcome-driven interventions by integrating private sector capacity into the national health system.
Case 2	Mr. B, who is a M40 Malaysian citizen.	Mr. B is enrolled in the NSHS scheme for himself and his household. His personal contribution is partially subsidized by the government.	Mr. B requires an elective gallbladder removal, but the earliest available surgery date in the public hospital is nine months away, which is far beyond the clinically accepted maximum of three months.	To ensure timely care, the government will utilize NSHS funds to contract this procedure to a private hospital, allowing Mr. B to receive his surgery within the recommended timeframe. Post-surgery, Mr. B will return to the public hospital for follow-up care and subsequently transition to a designated private GP for long-term monitoring and management. This GP, affiliated with the NSHS network, operates under the oversight of a linked government Family Medicine Specialist who is responsible for mentoring, auditing quality of care and clinical outcomes.	Mr. B’s case demonstrates how NSHS enables strategic coordination between public and private providers, ensuring timely access to surgery, continuity of care, and quality assurance across the entire treatment pathway.
Case 3	Mr. C, a T20 Malaysian citizen.	Mr. C contributes to both the NSHS and a private medical insurance plan.	Three months ago, he suffered a heart attack, and the initial treatment and follow-up were covered by his private insurer. However, that coverage ended after the standard 3-month period.	Rather than bearing the OOP costs, Mr. C seamlessly transitioned to NSHS for continued follow-up care at a public hospital.	Mr. C case demonstrates the critical value of NSHS as a complementary safety net, providing ongoing support when private insurance reaches its limit.
Case 4	Baby D, a 2-day-old neonate born in a government hospital.	Baby D’s family (T20) voluntarily opted in for NSHS coverage.	Baby D was born prematurely and urgently requires ventilatory support in a NICU. However, all NICU ventilators in the government hospital and nearby public facilities are at full capacity.	As a result, Baby D must be referred to a private NICU to receive life-saving care. Under the NSHS, the cost of this private NICU admission will be fully covered.	This case illustrates the critical role of NSHS in bridging service gaps, enabling timely access to essential care, and optimizing use of available healthcare resources across sectors.

Abbreviations: B40, the bottom 40% of income earners; M40, the middle 40% of income earners; T20, the top 20% of income earners; NSHS, National Social Health Scheme; GP, general practitioner; OOP, out-of-pocket; NICU, neonatal intensive care unit.

 The second revenue source is from the Malaysian Government, committing a fixed, increasing share of national GDP annually. Funds will continue to target public health, infrastructure, and workforce growth, which are critical to long-term health security and national productivity.^9^ Together with NSHS contributions, these streams will support a resilient, equitable, and sustainable healthcare financing system.

###  Contributions Towards National Social Healthcare Scheme

 An income-based contribution model (Figure) ensures that the healthcare fund grows progressively as salary increases. The government is to fully subsidise contributions for the B40, persons with disabilities, and pensioners. For the middle 40% of income earners (M40), the government will partially subsidise 60%, with the remaining 40% covered through individual co-contribution. The top 20% of income earners (T20) would self-finance their full contribution. For employed individuals in the M40 and T20 groups, employers will contribute 50% of individual total required contributions. Those who opt out of the NSHS will pay OOP for services under NSHS. Those who has both private insurance and NHSH will find that NSHS will be a complementary safety net when private insurance reaches its limit (Table: Case 3).

###  Launchpad for National Social Healthcare Scheme 

 Social Security Organization** (**SOCSO),^18^ which insures millions of private workers across all income levels, is well-positioned to launch the NSHS. While current contributions (0.5%–1.25%) cover only occupational risks, its established system, efficient claims process, and nationwide reach can support general healthcare. An added 0.75% healthcare contribution, assessed by actuaries, can ensure sustainability without overburdening contributors.

###  Strengthening Public-Private Partnership, Gatekeeping and Cost-Containment

 Although early-stage PPPs exist, they remain fully government-funded and is unsustainable. A well-designed PPP should tap private capacity without draining the NSHS fund. Cost containment can be achieved through strict gatekeeping and OOP charges for patients bypassing the system.^16^ In the NSHS model (Figure), public and private primary care providers serve as the first gatekeepers, delivering acute and preventive care, reducing unnecessary emergency visits, and managing referrals. Integrating private GPs into the NSHS will require coordinated collaboration, with Family Medicine Specialists providing mentorship to maintain consistent, high-quality care. Private GPs, contracted via the SOCSO–Malaysian Medical Association GP Section Committee, will be selected based on postgraduate qualifications, location, and population needs, ensuring wide coverage and consistent quality, with payment per Schedule 7 of the Private Healthcare Facilities and Services Act 1998.^19^

 Public hospitals act as the second gatekeeper, referring patients to private hospitals only when necessary and ensuring back-referrals. Hospital selection will be managed by a joint panel from the Association of Private Hospitals Malaysia and SOCSO, under the oversight of an independent regulatory body. Selection will prioritize Malaysian Society for Quality in Health-accredited providers agreeing to pre-set fee ceilings.

 Hospitals must use strong electronic health record systems and standardized coding to ensure complete data, enabling accurate diagnostic-related group (DRG) coding built on extensive casemix. Coders should be trained to ensure accuracy. Regular audits must be in place to detect errors and prevent system manipulation, with strict penalties and rigorous data checks to stop upcoding for higher payments.^20,21^ The government should also pool-purchase essential supplies and enforce restrictions on costly drugs, a critical measure for cost control and protecting limited resources.^16^

###  Strengthening Preventive Care Through Price-to-Risk Insurance Model for Existing Private Insurance

 Alongside the NSHS, private insurance should shift from uniform-based products to a price-to-risk model that rewards healthy lifestyles. Currently, most policies differ mainly by annual coverage limits, disadvantaging both individuals with higher health risks who face increased premiums or even denial of coverage, and those who maintain a healthier lifestyle, who see little to no reward. A more responsive model should offer lower premiums and incentives for quitting smoking, maintaining a healthy body mass index, or meeting metabolic health targets. This is a critical step forward in building a stronger preventive care within the healthcare ecosystem.

## Implementation Strategy

 Participation in the NSHS is key for risk pooling and sustainability, but public concerns must be addressed. Diversifying funding is politically sensible act, and it is vital for national health sustainability. Transitioning should be gradual, following a 7-phase plan with strong planning and stakeholder engagement to ensure success and avoid political backlash.

###  Phase 1: Feasibility Study (Timeline: 3 Months)

 The first step is a discreet, thorough feasibility study to assess Malaysia’s healthcare landscape, address hybrid system challenges, evaluate risks, and define the optimal NSHS and DRG structure for the country’s demographic and economic context. Early engagement of Bank Negara Malaysia, insurers, actuaries, risk experts, private providers, and Ministry of Health Malaysia (MoH) is crucial for sound financial governance. Payment mechanisms must be rigorously evaluated and monitored to prevent manipulation and protect care quality.

###  Phase 2: Winning the Hearts and Minds (Timeline: 3 Months)

 To secure public support, the government must run a targeted, sustained campaign across social and mainstream media, using consistent, data-driven, and emotionally engaging messaging backed by real-life examples.^22^ Communication must highlight the NSHS’s value for all income groups; comprehensive care for the B40; added coverage for the M40 and T20; and complementing private insurance. Early engagement with opinion leaders, medical professionals, influencers, and netizens will be critical for building trust. Public healthcare workers should be involved from the outset through inclusive consultations via a special task force, supported by capacity building, recognition of expertise, clear incentives, and accountability.

###  Phase 3: Pilot Program (B40) (Timeline: 1 Year)

 Once awareness is established, pilot the NSHS with fully subsidised groups (B40) to provide quality care in both public and private facilities while boosting political support. Start in low-volume regions to avoid straining high-pressure urban hospitals that are already under significant strain. Pilots should rigorously test the model, generate data, and refine the rollout strategy. Multiple rounds to be expected before national expansion as rushed implementation risks failure. Each pilot must deliver actionable insights to strengthen operational readiness and scalability.

###  Phase 4: Nationwide Rollout (Timeline: 2 Years)

 A phased national rollout should follow, backed by scaled infrastructure, workforce training, and ongoing public engagement. Clinical quality standards must be strictly enforced.

###  Phase 5: Continuous Monitoring and Evaluation (Timeline: 3rd Year Onwards)

 A robust monitoring and evaluation framework must be established from the start, with clear performance indicators, regular reporting, and independent audits for transparency and accountability.

###  Phase 6: Accountability and Governance (From Now)

 A strong, independent regulatory body separate from the MoH and Bank Negara Malaysia must be established to oversee NSHS implementation. This body will regulate, audit, and govern public and private healthcare providers and private health insurers. This is crucial to restore balance, protect patients, and ensure the system’s long-term sustainability. Public confidence relies on visible outcomes, which depend on disciplined governance.

###  Phase 7: Political Consideration (From Now)

 The government must acknowledge the critical dissatisfaction among healthcare workers and the public. Many recent proposals are seen as reactive, short-term fixes,^23^ further eroding trust and increasing frustration. The introduction of the NSHS may face resistance and scepticism, particularly if seen as another top-down directive. However, with strong political will and strategic framing, the NSHS offers not a political risk, but an opportunity for meaningful, lasting reform that tackles root causes. If implemented thoughtfully, the NSHS can restore confidence, enhance system performance, and prove the government’s genuine commitment to national health and future prosperity. Done right, it will be a legacy-defining achievement.

## Conclusion

 Malaysia must act decisively to prevent the impending collapse of its healthcare system. Adopting a hybrid healthcare financing model is not optional but is urgent and essential. By combining government subsidies, NSHS, DRG, and strong PPP, the country can create a resilient, equitable, and high-performing system that provides quality care for all. A key element of this transformation is the integration both public and private healthcare services to strengthen service delivery, improve access, and alleviate pressure on public facilities. With the right financial framework and political will, Malaysia can future proof its healthcare system and ensure health security for every citizen. The next step is to pilot the scheme among the fully government-funded B40 group to test its effectiveness.

## Acknowledgements

 We would like to express our sincere gratitude to the following individuals for their collaborative effort and invaluable expert input in the development of this concept paper: Associate Professor Dr. Mohd Fahmi Lukman, an Anaesthesiologist and Critical Care Consultant at National Defence University of Malaysia; Associate Professor Dr. Ambigga Devi S. Krishnapillai, a Family Medicine Consultant at National Defence University of Malaysia; Dr. Aida Jaffar a Family Medicine Consultant, at National Defence University of Malaysia; Dr. Maizatullifah Miskan, a Family Medicine Consultant at National Defence University of Malaysia; Dr. Siti Athirah Zafirah binti Abdul Rashid, a Health Economist Specialist at National Defence University of Malaysia and Mr. Nicholas Khaw, Head of Research and Co-Head of Private Markets at Khazanah Nasional Berhad, Malaysia.

## Ethical issues

 Not applicable.

## Conflicts of interest

 Authors declare that they have no conflicts of interest.
